# *Pseudomonas syringae* pv. *actinidiae* Type III Effectors Localized at Multiple Cellular Compartments Activate or Suppress Innate Immune Responses in *Nicotiana benthamiana*

**DOI:** 10.3389/fpls.2017.02157

**Published:** 2017-12-20

**Authors:** Sera Choi, Jay Jayaraman, Cécile Segonzac, Hye-Jee Park, Hanbi Park, Sang-Wook Han, Kee Hoon Sohn

**Affiliations:** ^1^Bioprotection Research Centre, Institute of Agriculture and Environment, Massey University, Palmerston North, New Zealand; ^2^Department of Life Sciences, Pohang University of Science and Technology, Pohang, South Korea; ^3^Plant Science Department, Plant Genomics and Breeding Institute and Research Institute of Agriculture and Life Sciences, College of Agriculture and Life Sciences, Seoul National University, Seoul, South Korea; ^4^Department of Integrative Plant Science, Chung-Ang University, Anseong, South Korea; ^5^School of Interdisciplinary Bioscience and Bioengineering, Pohang University of Science and Technology, Pohang, South Korea

**Keywords:** effector screening, avirulence, *Nicotiana benthamiana*, *Nicotiana tabacum*, hypersensitive response, virus-induced gene silencing, subcellular localization

## Abstract

Bacterial phytopathogen type III secreted (T3S) effectors have been strongly implicated in altering the interaction of pathogens with host plants. Therefore, it is useful to characterize the whole effector repertoire of a pathogen to understand the interplay of effectors in plants. *Pseudomonas syringae* pv. *actinidiae* is a causal agent of kiwifruit canker disease. In this study, we generated an *Agrobacterium*-mediated transient expression library of YFP-tagged T3S effectors from two strains of *Psa*, *Psa*-NZ V13 and *Psa*-NZ LV5, in order to gain insight into their mode of action in *Nicotiana tabacum* and *N. benthamiana*. Determining the subcellular localization of effectors gives an indication of the possible host targets of effectors. A confocal microscopy assay detecting YFP-tagged *Psa* effectors revealed that the nucleus, cytoplasm and cell periphery are major targets of *Psa* effectors. *Agrobacterium*-mediated transient expression of multiple *Psa* effectors induced HR-like cell death (HCD) in *Nicotiana* spp., suggesting that multiple *Psa* effectors may be recognized by *Nicotiana* spp.. Virus-induced gene silencing (VIGS) of several known plant immune regulators, *EDS1*, *NDR1*, or *SGT1* specified the requirement of SGT1 in HCD induced by several *Psa* effectors in *N. benthamiana*. In addition, the suppression activity of *Psa* effectors on HCD-inducing proteins and PTI was assessed. *Psa* effectors showed differential suppression activities on each HCD inducer or PTI. Taken together, our *Psa* effector repertoire analysis highlights the great diversity of T3S effector functions *in planta*.

## Introduction

Plants have developed two layers of defense to protect themselves from invading pathogens. The first layer, PAMP-triggered immunity (PTI), is facilitated by pattern recognition receptors (PRRs) that recognize conserved microbial molecules termed pathogen associated molecular patterns (PAMPs). Recognition of PAMPs such as bacterial flagellin, lipopolysaccharide or elongation factor Tu (EF-Tu) allows plants to mount immunity against a broad range of pathogens (Bigeard et al., 2015). Nevertheless, successful bacterial pathogens can deliver a suite of effector proteins via a type III secretion system (T3S) into host cells to dampen PTI. In turn, plants have evolved a second layer of defense to recognize specific effectors and induce effector-triggered immunity (ETI). This is often accompanied by rapid programmed cell death termed a hypersensitive response (HR) at the site of infection. As a result, in most of cases, plants can survive despite constant exposure to a wide range of pathogens.

Bacterial effectors, which primarily function to suppress immunity, have diverse biochemical functions and host virulence targets. Appropriate subcellular localization within the host cell is required for effectors to properly reach their targets to function (Hicks and Galán, 2013). Many studies have demonstrated the vast array of effector biochemical functions and subcellular localizations. For example, the type III effector AvrPto from *P. syringae* pv. *tomato* (*Pto*) is localized to the plasma membrane where it targets the membrane-associated PRRs FLS2 (FLAGELLIN SENSING 2) and EFR (ELONGATION FACTOR Tu RECEPTOR) thus inhibiting their phosphorylation, leading to reduced PTI responses (Xiang et al., 2008). The plasma membrane localized effectors AvrRpm1 from *P. syringae* pv. *maculicola* and AvrB from *P. syringae* pv. *glycinea* both require membrane localization by myristoylation for targeting the host protein RIN4 as well as avirulence functions (Nimchuk et al., 2000; Mackey et al., 2002). Another *Pto* effector, HopAI1, localizes to the cytoplasm where it inactivates MPK3 and MPK6, that play a key role in PTI signaling, via its phosphothreonine lyase activity (Zhang et al., 2007). A number of *P. syringae* effectors including HopI1, HopN1, HopK1, and AvrRps4 target the chloroplast to suppress immunity (Jelenska et al., 2007; Rodriguez-Herva et al., 2012; Li et al., 2014). Moreover, the *Pto* DC3000 effector HopM1 localizes to the *trans*-Golgi network and interacts with host ADP-ribosylation factor guanine nucleotide exchange factor, AtMIN7, to suppress vesicle-trafficking during immune responses (Nomura et al., 2011). Bacterial effectors do not interfere solely with PTI; some bacterial effectors have been shown to suppress ETI responses. Recently, the *Pto* DC3000 effector HopD1 was reported to suppress ETI by localizing to the endoplasmic reticulum to interact with the host membrane-tethered transcription factor NTL9 (Block et al., 2014). Some fascinating mechanisms of effector activity within the nucleus have been demonstrated, such as the *Ralstonia* effector PopP2 that contains a nuclear localization signal (NLS) and targets WRKY transcription factors in order to disable defense signaling (Sarris et al., 2015). Interestingly, Arabidopsis has evolved the paired immune receptors, RRS1 and RPS4, that interact with PopP2 via the RRS1 C-terminal decoy WRKY domain to trap PopP2 and activate ETI responses (Williams et al., 2014). The host nucleus is a key effector target for the disruption of immune responses. A recent study of the localization of ∼50 RxLR effectors from the Arabidopsis downy mildew oomycete pathogen, *Hyaloperonospora arabidopsidis* (*Hpa*) Emoy2, revealed that the majority of these effectors were found to be localized at membranes or in the nucleus (Caillaud et al., 2012a,b). Furthermore, Caillaud et al. (2012a) identified that the tonoplast-localized *Hpa* effector HaRxL17 functions as a virulence effector during infection. It is clear that understanding the subcellular localization of pathogen-derived effectors is of great importance to better understand their virulence or avirulence mechanisms.

The co-evolutionary arms race between pathogen effectors and their corresponding plant intracellular immune receptors has shaped the highly diversified repertoire of both (Jones and Dangl, 2006). The majority of characterized plant intracellular immune receptors are nucleotide-binding and leucine-rich repeat receptors (NLRs). NLR proteins carry a variable amino-terminal region with either a CC (coiled-coil) or TIR (toll-interleukin1 receptor-like) domain that is generally involved in activating downstream defense signaling (Meyers et al., 2003; Cui et al., 2015). Only a few immune regulators have been characterized for their role in NLR-mediated signaling. Typically, the lipase-like protein EDS1 (ENHANCED DISEASE SUSCEPTIBILITY 1) is required for immune signaling pathways initiated by TIR-NLRs (TNLs), whereas integrin protein NDR1 (NON-RACE SPECIFIC DISEASE RESISTANCE 1) is required downstream of CC-NLRs (CNLs) (Aarts et al., 1998). Moreover, SGT1 (SUPPRESSOR OF THE G2 ALLELE OF *SKP1*) forms a complex with the molecular chaperone HSP90 (HEAT SHOCK PROTEIN 90) to maintain proper folding of NLR proteins and are often required for NLR functions (Austin, 2002; Takahashi et al., 2003). Therefore, investigating the requirement of these regulators in effector-triggered immunity would help better understand *in planta* functions of effectors.

A few effectors have been shown to have diverse and complex functions *in planta*. Previous studies have shown that the *Pto* DC3000 effector HopAB3 is able to suppress ETI and PTI (Abramovitch et al., 2006; Goehre et al., 2008; Guo et al., 2009). Moreover, suppression of *P. syringae* pv. *syringae* effector HopA1-triggered HR in Arabidopsis by co-delivered effectors identified many ETI-suppressing effectors from *Pto* DC3000 that were also able to suppress *P. syringae* pv. *maculicola* effector AvrRpm1-triggered ETI (Guo et al., 2009). In the same study, some of these ETI-suppressing effectors also suppressed PTI responses. Similarly, a large number of effectors from the oomycete pathogen *Phytophthora sojae* were found to suppress PTI and ETI (Wang et al., 2011). Upon infection, these PTI/ETI-suppressing effectors would greatly affect the plant–pathogen interaction.

*Pseudomonas syringae* pv. *actinidiae* (*Psa*) causes bacterial canker disease in kiwifruit. Since the 2008 outbreak of highly virulent *Psa* (*Psa-*V) in Italy, *Psa*-V has spread worldwide, including Chile (2010) and New Zealand (2010). *Psa-*V is phylogenetically distinct from the low virulent strain (*Psa-*LV) (McCann et al., 2013). These authors suggested that these distinctive clades are the result of various gene-shifting processes derived from different source populations, hinting at the likely probability of new virulence emergence in *Psa* populations. In fact, evidence suggests selection driving horizontal gene transfer in a *Psa*-V strain to gain copper resistance from a local *Psa*-LV strain (Colombi et al., 2017). Considering the active transfer and conversion events of virulence effector genes in *Psa* source populations, research efforts on the effector gene pools in geographically co-existing strains such as *Psa-*LV and *Psa-*V would be useful.

In this study, we sought to characterize the effector repertoire of one representative strain from each clade present in New Zealand, namely the virulent strain *Psa* NZ V-13 and the low virulence strain *Psa* NZ LV-5 (hereafter, *Psa* V13 and *Psa* LV5, respectively), both isolated from diseased orchards in the Bay of Plenty region, New Zealand (Chapman et al., 2012). We used *Agrobacterium-*mediated transient transformation to test subcellular localization and cell death-inducing activity of *Psa* effectors in non-host plants *Nicotiana benthamiana* and *N. tabacum*. We showed that *Psa* effectors localized to different plant cell compartments, presumably to interfere with multiple plant defense-related processes. We also found that multiple *Psa* effectors induced HR-like cell death and, using virus-induced gene silencing (VIGS), identified the requirement of known plant immunity regulators for effector-induced cell death. In addition, we demonstrated that several *Psa* effectors suppressed cell death triggered by other *Psa* effectors. We expect that the various effector characteristics found in this study associated with their putative biological functions will help to predict roles of these effectors *in planta* and aid in the development of bacterial canker-resistant kiwifruit.

## Results

### Construction of *Pseudomonas syringae* pv. *actinidiae* Type III Effector Libraries

To characterize the type-III secreted effector (T3E) repertoire of *Psa*, we cloned effectors from two *Psa* strains, V13 and LV5. *Psa* V13 and LV5 strains carry 38 and 26 T3Es, respectively, based on the computational prediction using their genome sequences (McCann et al., 2013; Templeton et al., 2015). Based on this, we selected a total of 48 T3Es for further study using the following criteria: (i) all V13 or LV5 specific T3Es, (ii) only V13 alleles for T3Es in both V13 and LV5 that shared more than 90% amino acid identity, or (iii) both V13 and LV5 alleles for T3Es that shared less than 90% amino acid identity (**Table 1**). Based on the protein sequence identity to *P. syringae* homologs, *Psa* V13 effectors that are predicted to be significantly truncated (*hopA1, hopW1, hopAA1-1*, and *hopAA1-2*) were excluded (**Table 1**). Only one allele of *Psa* V13 for duplicated effectors *hopBB1* (*hopBB1-1/hopBB1-2*, 93.6% identity) and *hopAM1* (*hopAM1-1/hopAM1-2*, 100% identity) were functionally analyzed. Additionally, both alleles of *hopAY1* were analyzed because the predicted peptide sequence of *Psa* V13 allele was only 77 amino acids shorter than that of LV5. In order to generate broad host-range plasmid (pBBR 1MCS-5) constructs for *Pseudomonas*-delivery and binary plasmid constructs for *in planta* transient expression of the 48 selected *Psa* T3Es, we used the Golden Gate cloning method (Engler et al., 2008). Briefly, each T3E sequence was divided into several modules roughly of 1 kb size and each module was amplified by polymerase chain reaction (PCR) using primers with flanking *Bsa*I restriction enzyme sites. These amplicons were cloned into the Golden Gate compatible entry vector pICH41021. The number of modules for each effector and their 4 bp *Bsa*I overhangs are listed in Supplementary Table S1. All modules for a given effector were then assembled with a C-terminal 6xHA-tag module into the Golden Gate-compatible derivative of the broad host-range vector pBBR1MCS-5, which carries the bacterial *avrRps4* promoter (Jayaraman et al., 2017); or with a C-terminal YFP-tag module into the binary vector pICH86988 under control of a cauliflower mosaic virus (CaMV) 35S promoter for functional analysis.

**Table 1:** Type III secreted effectors from *Psa* V13 and LV5 used in this study.

Effectors	*Psa*-	*Psa*-	AA	Predicted
	V13	LV5	identity	protein size
			(%)	(kDa)
AvrB4-1	o		NA	36.0
AvrD1	o			34.6
AvrE1	o	o	97.1	195.3
AvrPto5	o			17.2
AvrRpm1	o			24.9
HopA1	o^§^	o		22 (V13)/42.2 (LV5)
HopD1	o			34.6
HopE1		o		24.1
HopF1		o		21.9
HopF4b (HopF2)	o			22.0
HopH1	o			24.3
HopI1	o			49.3
HopM1	o			75.9
HopN1	o	o	99.6	38.7
HopO1		o		32.6
HopQ1	o			48.8
HopR1	o	o	99.0	210.3
HopS1		o		19.9
HopS2	o	o	94.9	18.7
HopT1		o		41.5
HopW1	o^§^	o		40.8 (V13)/82.8 (LV5)
HopX1		o		40.6
HopX2		o		38.0
HopX3	o			40.9
HopY1	o	o		30.9
HopZ3	o			45.1
HopAA1	o^§^	o		21.8 (V13)/50.4 (LV5)
HopAB3		o		62.6
HopAE1	o	o	99.8	126.6
HopAF1	o	o		23.4
HopAF1-2		o		30.9
HopAG1		o		77.6
HopAH1	o	o	91.2	42.8
HopAI1^†^	o	o	81.6	29.7
HopAM1-1	o			31.3
HopAO2	o			38.1
HopAR1		o		28.5
HopAS1	o	o	98.9	149.0
HopAU1	o			88.2
HopAV1	o			123.5
HopAW1	o			24.9
HopAY1^‡^	o	o	91.9	27.1(V13)/35.4(LV5)
HopAZ1	o	o	99.5	24.7
HopBB1-1(2)	o			30.7
HopBN1	o			56.8


*^§^Effectors highly truncated in *Psa* V13 indicating a pseudogene.*

*^†^Amino acid identity between alleles from *Psa* V13 and LV5 lower than 90%.*

*^‡^*Psa* V13 version has shorter amino acid sequence.*

### Subcellular Localization of *Psa* Effectors in *Nicotiana benthamiana* Leaf Cells

**FIGURE 1:**
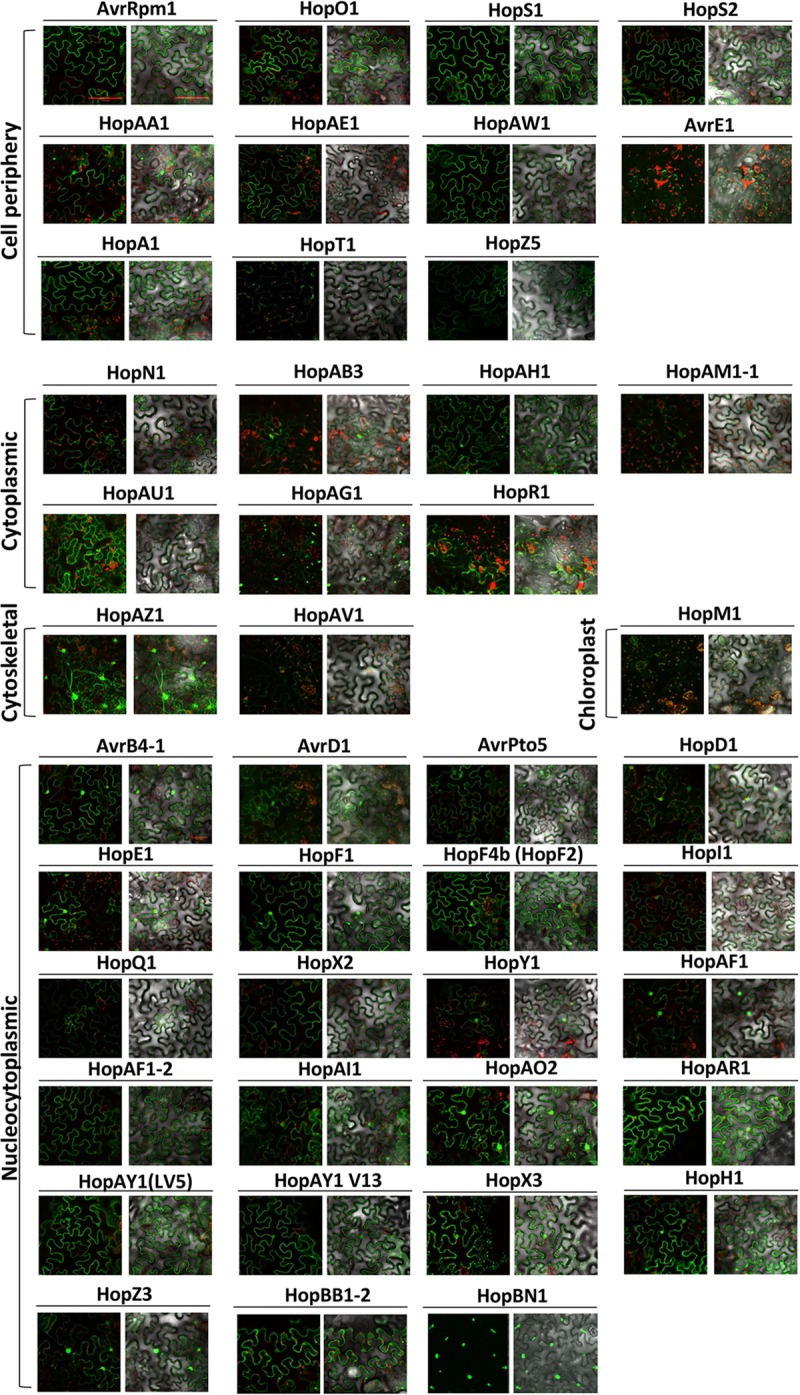
Subcellular localization of *Psa* effectors in *Nicotiana benthamiana.* Four–five week-old *N. benthamiana* leaf cells were infected with *Agrobacterium* AGL1 carrying C-terminally YFP-tagged *Psa* effectors for transient protein expression. At 2 days post infection (dpi), 8 mm diameter leaf disks were sampled and viewed using confocal microscopy. YFP fluorescence was excited at 488 nm with a 20 mW Argon laser and captured in the emission range between 500 and 530 nm. HopM1 YFP signal was determined by a sum-of-squares Z-projection. Chloroplast auto-fluorescence was detected between 600 and 680 nm. This experiment was repeated twice with similar results.

Determining the subcellular localization of effectors can suggest clues about their mode of action during infection. Several studies have highlighted the importance of localization of effectors in a particular cellular compartment for their *in planta* function (Lewis et al., 2008; Schornack et al., 2009; Sohn et al., 2014). To investigate the subcellular localization of *Psa* effectors, we expressed YFP-tagged *Psa* effectors in *N. benthamiana* leaf cells using *Agrobacterium*-mediated transient transformation (hereafter agroinfiltration). To visualize effector-YFP proteins, confocal laser scanning microscopy was undertaken at 48 h after agroinfiltration. The subcellular localization of all tested *Psa* effectors are shown in **Figure 1** (larger images for the localization representatives are in Supplementary Figure S2). Interestingly, approximately half of the *Psa* effectors (23 effectors) were localized in the nucleus and cytoplasm. Only one effector, HopBN1, exclusively localized in the nucleus (**Figure 1** and Supplementary Figure S2). HopBB1-2, localized in the nucleus and cytoplasm as well as subnuclear foci (**Figure 1** and Supplementary Figure S2). We identified seven effectors (HopN1, HopR1, HopAB3, HopAG1, HopAH1, HopAM1-1, and HopAU1) that localized to the cytoplasm but were excluded from the nucleus (**Figure 1** and Supplementary Figure S2). Among these, HopAG1 showed punctate localization. HopM1 localized to the chloroplasts, while HopAV1 and HopAZ1 localized to the cytoplasm but in strands resembling the cytoskeleton (**Figure 1** and Supplementary Figure S2). Eleven effectors localized to the cell periphery (largely absent from cytoplasmic strands), and two of them (HopT1 and AvrE1) showed punctate localization (**Figure 1**) (Jayaraman et al., 2017). However, we could not determine the subcellular localization of HopAS1, HopW1, and HopX1 in our experimental conditions. Notably, these results suggest that *Psa* bacterial effectors are localized and, therefore, function at several distinct cellular compartments.

**FIGURE 2:**
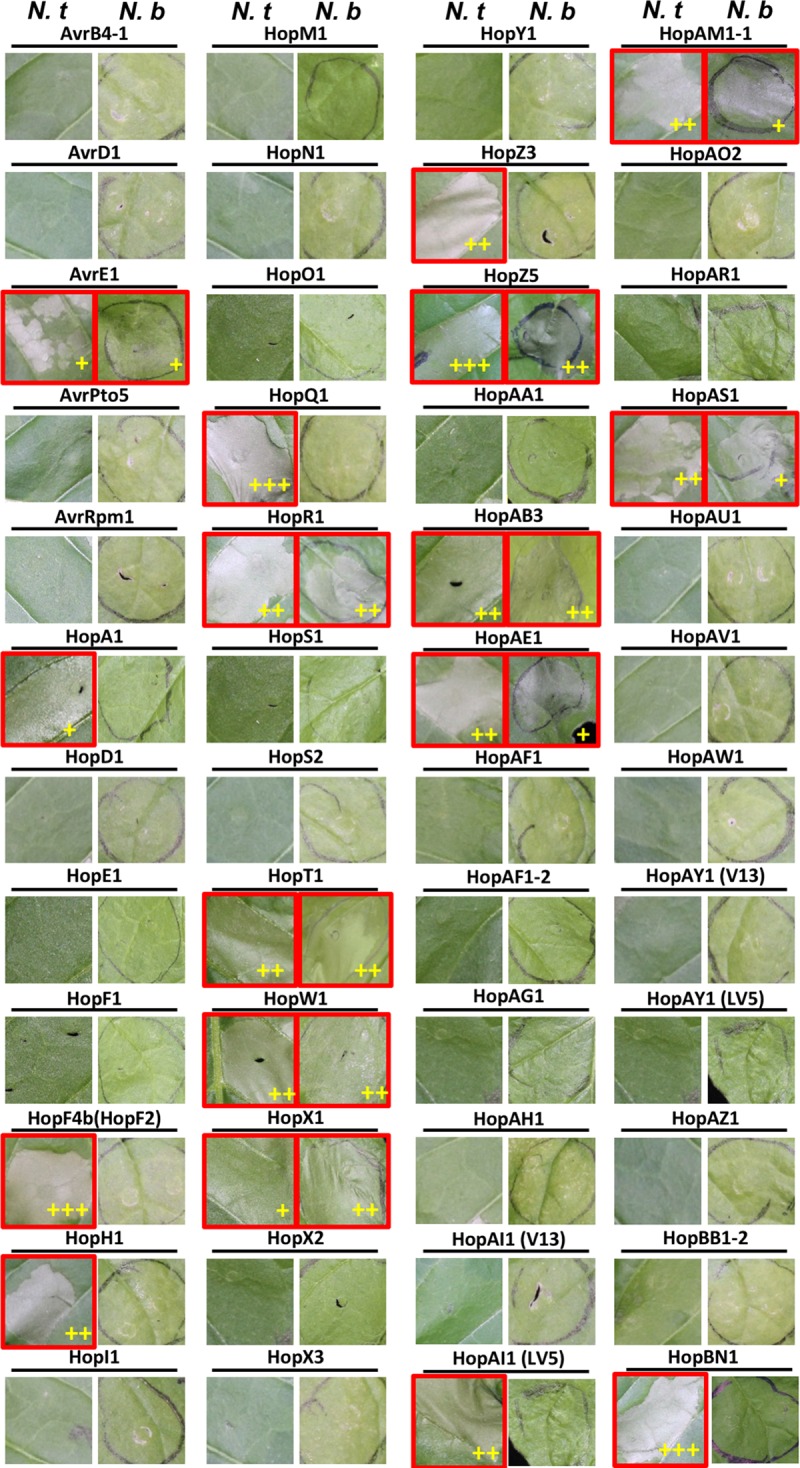
Multiple *Psa* effectors trigger HR-like cell death in *Nicotiana* spp. Four–five weeks-old *N. benthamiana* and *N. tabacum* plants were infiltrated with *Agrobacterium tumefaciens* AGL1 strains carrying C-terminally YFP-tagged *Psa* effector constructs. Photographs of the leaves showing HR-like cell death (HCD) were taken at 3 dpi. Leaf images showing HCD are indicated with a red border. (+), (++), or (+++) indicate the strength of the HCD phenotype as measured by ion conductivity (Supplementary Figures S3, S4). This experiment was conducted at least three times with similar results.

In order to validate the subcellular localization of effector-YFP fusion proteins, immunoblot analysis was conducted using total protein extracts from agroinfiltrated *N. benthamiana* leaves (Supplementary Figure S1). Out of 48 effectors, 38 were confirmed for protein expression by anti-GFP immunoblots (Supplementary Figure S1) (Jayaraman et al., 2017). Of the 10 effectors that could not be detected, six (AvrE1, HopR1, HopT1, HopW1, HopAM1-1, and HopAS1) triggered cell death in *N. benthamiana* (**Figure 2**). The remaining 4 effectors (HopI1, HopBN1, AvrD1, and HopAV1) could not be detected despite the lack of strong cell death response. In contrast, HopX1, which also triggered cell death, was detected in the immunoblot but could not be localized via confocal microscopy. The subcellular localization of AvrE1, HopR1, HopBN1, HopI1, HopAM1-1, HopAV1, and HopT1 was detected by confocal microscopy but protein expression was not validated by immunoblot analysis.

### Multiple *Psa* Effectors Induce HR-Like Cell Death in *Nicotiana* spp.

Multiple *P. syringae* effectors have been shown to induce HR-like cell death (HCD) in *Nicotiana* (Vinatzer et al., 2006; Wei et al., 2007; Wroblewski et al., 2009). To identify *Psa* T3Es that induce HCD in *N. benthamiana* or *N. tabacum*, we transiently expressed each *Psa* effector using agroinfiltration. Surprisingly, 17 *Psa* T3Es induced HCD in *N. tabacum*. Of these, 10 *Psa* T3Es also triggered HCD in *N. benthamiana* (**Figure 2** and Supplementary Figures S3, S4) (Jayaraman et al., 2017). Interestingly, none of the tested *Psa* T3Es triggered *N. benthamiana-*specific HCD. In *N. tabacum*, 17 T3Es [AvrE1, HopA1, HopF4b, HopH1, HopQ1, HopR1, HopT1, HopW1, HopX1, HopZ3, HopAB3, HopAE1, HopAI1 (LV5), HopBN1, HopAM1-1, HopZ5 and HopAS1] induced HCD in 3–4 days. The intensity of HCD was quantified by ion conductivity measurement at different time points (Supplementary Figure S3). Fourteen *Psa* T3Es triggered a full collapse of infiltrated leaf tissue while 3 effectors (AvrE1, HopX1, and HopA1) triggered a slower/weaker HCD (**Figure 2** and Supplementary Figure S3). *Psa-*effector triggered HCD was assessed similarly for *N. benthamiana*, and 6 T3Es (HopR1, HopW1, HopX1, HopAB3, HopZ5, and HopT1) induced strong HCD while 4 T3Es (AvrE1, HopAM1-1, HopAE1, and HopAS1) induced a slower/weaker response (**Figure 2** and Supplementary Figure S4).

Natural sequence diversity in the T3E repertoire of *P. syringae* strains can cause variation in their ability to induce HCD (Ma et al., 2006). In order to identify if allelic sequence variation between *Psa* T3Es and their homologs found in other *P. syringae* strains caused an altered HCD phenotype in *Nicotiana*, we compared their amino acid sequence identity and HCD-inducing ability based on published literature (**Table 2**). In total, 15 *P. syringae* homologs of *Psa* T3Es that were previously tested for HCD induction in *N. tabacum* and/or *N. benthamiana* were included in our comparison. Phylogenetic analysis showed that *Pto* DC3000 is closely related to *Psa* and 23 T3Es are conserved between these strains (Butler et al., 2013). We selected 10 *Pto* DC3000 T3Es that triggered HCD, or had a *Psa* homolog that triggered HCD in *Nicotiana* spp., for comparison (**Table 2**) (Wei et al., 2007; Wroblewski et al., 2009). In addition to *Pto* DC3000, 3 T3E homologs from *P. syringae* pv. *syringae* (*Psy*) B728a, one from *P. cannabina* pv. *alisalensis* ES4326 and one from *P. syringae* pv. *tomato* (*Pto*) T1 were analyzed (Lin et al., 2006; Robert-Seilaniantz et al., 2006; Vinatzer et al., 2006; Wroblewski et al., 2009).

**Table 2:** Comparison of HR-like cell death (HCD) phenotypes induced by *Psa* effectors and their *Pseudomonas syringae* homologs.

*Psa* effector	*N. benthamiana*/*N. tabacum*	Homologs	Source strain	*N. benthamiana*/*N. tabacum*	AA identity (%)	Reference
AvrE1	+/+	AvrE1	*Pto* DC3000	+/NA	94.1	1^∗^
HopA1	-/+	HopA1	*Pto* DC3000	-/NA	93.9	1
HopF4b	-/+	HopF2	*Pto* DC3000	-/+	57.6	1,2
HopF1	-/-				47.8	
HopH1	-/+	HopH1	*Pto* DC3000	-/NA	97.7	1
HopM1	-/-	HopM1	*Psy* B728a	+/+	71.9	3
HopQ1	-/+	HopQ1-1^a^	*Pto* DC3000	-/NA	99.1	1,4
HopR1	+/+	HopR1	*Pto* DC3000	-/NA	95.9	1
HopT1	+/+	HopT1-1^b^	*Pto* DC3000	+/NA	70.4	1,4
HopW1	+/+	HopW1-1	*Pcal* ES4326	+/NA	77.8	4
HopX1	+/+	HopX1	*Pto* DC3000	+/NA	73.7	1
HopZ3	-/+	HopZ3	*Psy* B728a	-/+	72.1	3
HopAA1	-/+	HopAA1-1	*Pto* DC3000	+/NA	93.8	1
HopAB3	+/+	AvrPtoB (HopAB3)	*Pto* T1	-/NA	83.5	5
HopAE1	+/+	HopAE1	*Psy* B728a	+/+	73.6	3
HopAM1-1	+/+	HopAM1-1^c^	*Pto* DC3000	-/NA	98.9	1


*^a^*Pto* DC3000 HopQ1-2 is highly truncated (Buell et al., 2003).*

*^b^*Psa* HopT1 shares 96.9% of amino acid sequence identity with *Pto* DC3000 HopT1-2.*

*^c^Both *Psa* and *Pto* DC3000 have two identical copies of HopAM1 (HopAM1-1/HopAM1-2).*

*NA, not available; *Pto*, *Pseudomonas syringae* pv. *tomato; Pcal*, *Pseudomonas cannabina* pv. *alisalensis; Psy*, *Pseudomonas syringae* pv. *syringae.**

*^1^(Wei et al., 2007), ^∗^Effector-triggered cell death was assessed by *Pseudomonas* type III secretion in this literature.*

*^2^(Robert-Seilaniantz et al., 2006).*

*^3^(Vinatzer et al., 2006).*

*^4^(Wroblewski et al., 2009).*

*^5^(Lin et al., 2006).*

**FIGURE 3:**
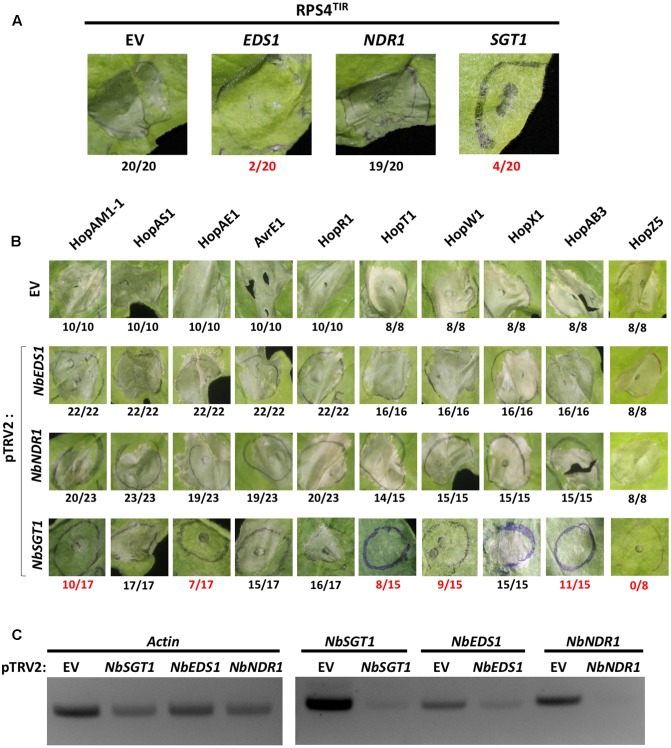
*NbSGT1* is partially required for HR triggered by several *Psa* effectors. **(A)** RPS4^TIR^-triggered HCD in *EV*–, *NbEDS1*–, *NbNDR1*–, or *NbSGT1*–silenced *N. benthamiana* plants. Two-week old *N. benthamiana* plants were infiltrated with mixture of *A. tumefaciens* AGL1 strains carrying pTRV1 or pTRV2 construct as indicated in section “Materials and Methods.” Four weeks later, plants were agroinfiltrated to express the RPS4 TIR domain (RPS4^TIR^). RPS4^TIR^-induced HCD was scored at 4 dpi. The number of HCD-showing patches out of total infiltrated patches are indicated under each panel. Numbers labeled in red indicate a significant reduction of HCD. **(B)** *Psa* effector-triggered HCD in *NbEDS1*–, *NbNDR1*–, and *NbSGT1*–silenced *N. benthamiana* plants. VIGS and agroinfiltration to induce HCD are indicated in **(A)**. *Psa* effector-induced HCD was scored at 4 dpi. The number of HCD-showing patches out of total infiltrated patches are indicated under each panel. Numbers labeled in red indicate a significant reduction of HCD. **(C)** Semi-quantitative PCR amplification of silenced genes in VIGS plants. 8 mm diameter leaf disks were harvested from VIGSed plants, total RNA was extracted by Trizol RNA method and cDNA was synthesized. Using cDNA as PCR template, partial fragments of *Actin* (control) and VIGSed genes were amplified with gene-specific primers.

Many *P. syringae* homologs showed similar phenotypes to their *Psa* counterparts in triggering HCD in *N. benthamiana* such as AvrE1, HopF4b, HopT1, HopW1, HopX1, HopAA1, and HopAE1 (**Table 2**). In *Pto* DC3000, there are two copies of *hopT1* (indicated as *hopT1-1* and *hopT1-2*). *Psa* HopT1 shares the highest amino acid sequence identity with *Pto* DC3000 HopT1-2 (96.9%). Despite this, we compared *Psa* HopT1 to *Pto* DC3000 HopT1-1 (70.4%) because the HCD phenotype was only tested for HopT1-1 (Wei et al., 2007; Wroblewski et al., 2009). Regardless, both *Psa* HopT1 and *Pto* DC3000 HopT1-1 triggered HCD in *N. benthamiana*. Conversely, there are several T3E homolog pairs that show differing phenotypes despite high amino acid sequence identity. For example, *Pto* T1 HopAB3 shares high amino acid identity with *Psa* HopAB3 (84.5%) (Lin et al., 2006). However, unlike HopAB3 from *Pto* T1, *Psa* HopAB3 induced HCD in both *N. benthamiana* and *N. tabacum.* In addition, *Pto* DC3000 T3Es HopAA1-1 and HopR1 are highly similar to *Psa* HopAA1 and HopR1 (93.8 and 95.9% amino acid identity, respectively) but only *Pto* DC3000 HopAA1-1 and *Psa* HopR1 induced HCD in *N. benthamiana* (**Figure 3**). *Pto* DC3000 HopQ1-1 and *Psa* HopQ1 share high amino acid sequence identity (99.1%) and they both trigger strong HCD in *N. tabacum* (**Table 2**). In contrast, despite relatively low amino acid sequence identity (57.6%), *Pto* DC3000 HopF2 and its *Psa* homolog HopF4b triggered HCD in *N. tabacum* while *Psa* HopF1 did not (**Table 2**). Taken together, our data indicate that natural sequence variation might have caused loss or gain of HCD-inducing activity of *Psa* effectors.

### HR-Like Cell Death Induced by Several *Psa* Effectors Requires *SGT1* in *Nicotiana benthamiana*

Knocking down gene expression of key components of plant immunity by VIGS has been widely used to investigate the involvement of defense components in plant immunity (Liu et al., 2002; Peart et al., 2002; Oh et al., 2014). To test the requirement of general immunity regulators for *Psa* effector-triggered HCD, we applied VIGS to generate *N. benthamiana* plants silenced for *EDS1* (*pTRV2:NbEDS1*), *NDR1* (*pTRV2:NbNDR1*), or *SGT1* (*pTRV2:NbSGT1*) together with control plants (*pTRV2:EV*). Two alleles of *NbEDS1* have been reported, *NbEDS1a* and *NbEDS1b*, and our VIGS construct was designed to target *NbEDS1a* as *NbEDS1b* is likely a pseudogene (Adlung et al., 2016; Ordon et al., 2017). Hereafter, *NbEDS1a* is referred to as *NbEDS1*. First, in order to validate our experimental conditions, HCD induced by agroinfiltration of the RPS4 TIR domain (RPS4^TIR^) was tested in *N. benthamiana* plants VIGSed for EV, *NbEDS1*, *NbNDR1*, or *NbSGT1*. RPS4^TIR^ induced HCD in EV– or *NbNDR1*–silenced but not in *NbEDS1*– or *NbSGT1*–silenced plants, consistent with previous results (**Figure 3A** and **Table 3**) (Zhang et al., 2004; Swiderski et al., 2009). Next, 10 T3Es (HopAM1-1, HopAS1, HopAE1, AvrE1, HopR1, HopT1, HopW1, HopX1, HopZ5, and HopAB3) that triggered HCD in *N. benthamiana* were agroinfiltrated in *NbEDS1*–, *NbNDR1*– or *NbSGT1*–silenced plants. Interestingly, *Psa* effector-triggered HCD in *NbEDS1*– or *NbNDR1*–silenced plants was comparable to EV-silenced plants (**Figure 3B**). However, in *NbSGT1*–silenced plants, 6 T3Es, HopAM1-1, HopAE1, HopT1, HopW1, HopZ5, and HopAB3 were significantly affected in their ability to trigger HCD (**Figure 3B** and **Table 3**) (Jayaraman et al., 2017). In contrast, HopAS1-, HopX1-, AvrE1-, and HopR1-triggered HCD was not affected by *NbSGT1*–silencing. To further analyze the efficiency of VIGS, semi-quantitative RT-PCR was conducted. As a result, the level of *NbEDS1*, *NbNDR1*, and *NbSGT1* transcripts were significantly reduced in the respective silenced plants compared to EV-silenced control plants indicating that these genes were sufficiently silenced (**Figure 3C**). However, we noticed that unlike *NbNDR1*, *NbEDS1*-, or *NbSGT1*-silenced plants showed significantly reduced but not completely eliminated transcript levels of silenced genes. Taken together, these results suggest that *SGT1* plays a key role in HCD induced by several *Psa* effectors.

**Table 3:** Summary of *Nicotiana* HCD-triggering *Psa* effectors.

*Psa* effector	*In vivo* localization	HR-like cell death in *Nicotiana*		Requirement of defense regulators HR-like cell death in *N. benthamiana*
			
		*N. tabacum*	*N. benthamiana*	
AvrE1	Cell periphery	+	+	-
HopA1	Cell periphery	+	-	NA
HopF4b	Nucleus and cytoplasm	+	-	NA
HopH1	Nucleus and cytoplasm	+	-	NA
HopQ1	Nucleus and cytoplasm	+	-	NA
HopR1	Cytoplasm	+	+	-
HopT1	Cell periphery	+	+	*SGT1*
HopW1	Not localized	+	+	*SGT1*
HopX1	Not localized	+	+	-
HopZ3	Nucleus and cytoplasm	+	-	NA
HopAB3	Cytoplasm	+	+	*SGT1*
HopAE1	Cell periphery	+	+	*SGT1*
HopAI1 (LV5)	Nucleus and cytoplasm	+	-	NA
HopAM1-1	Cytoplasm	+	+	*SGT1*
HopAS1	Not localized	+	+	-
HopBN1	Nucleus	+	-	NA


*+, cell death.*

*-, no cell death.*

*NA, not available.*

### Multiple *Psa* Effectors Suppress Effector-Triggered Cell Death in *N. benthamiana*

**FIGURE 4:**
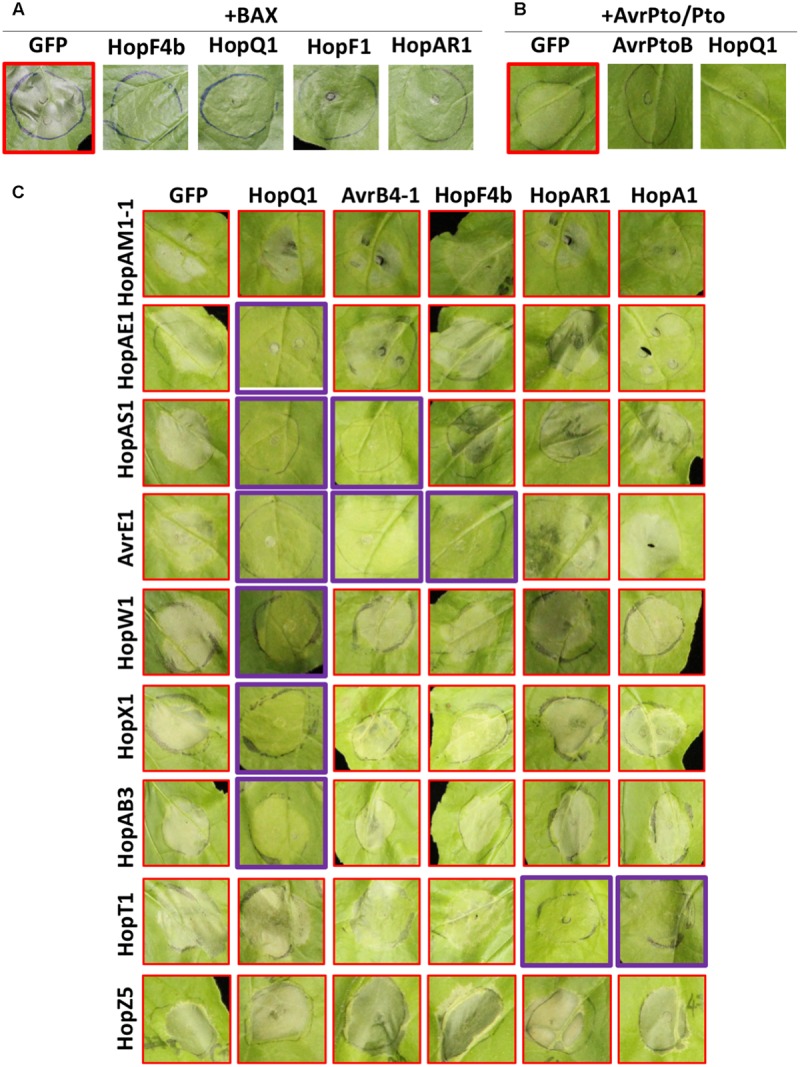
Multiple *Psa* effectors suppress HCD triggered by different effectors in *N. benthamiana.* **(A)** *Psa* effectors suppress BAX-induced HCD in *N. benthamiana. Agrobacterium* strains were mixed in a 1:4 ratio (BAX:*Psa* effector or GFP). **(B)** *Psa* effectors suppress AvrPto/Pto-induced HCD in *N. benthamiana. Agrobacterium* strains were mixed in 1:1:1 ratio (AvrPto:Pto:*Psa* effector or GFP). **(C)** Suppression of *Psa* effector-induced HCD in *N. benthamiana. Agrobacterium* strains were mixed in a 1:4 ratio (HCD-inducing effector:HCD-suppressing effector). Four–five week-old *N. benthamiana* leaves were agroinfiltrated and HCD was photographed at 3 **(A,B)** or 5 **(C)** dpi. This experiment was repeated more than three times with similar results.

Some pathogen effectors suppress ETI to enable pathogen proliferation. In order to investigate if *Psa* effectors can suppress ETI, we conducted an agroinfiltration assay of HCD-inducing proteins with or without *Psa* effectors in *N. benthamiana*. Bcl-2-associated X (BAX) is an animal pro-apoptotic regulator that induces HCD in plant cells when overexpressed (Lacomme and Santa Cruz, 1999). As previously shown, agroinfiltration of BAX with a GFP control induced strong HCD in *N. benthamiana* leaf cells (**Figure 4A**). Interestingly, several *Psa* effectors, HopF4b, HopQ1, HopF1, and HopAR1, suppressed BAX-induced HCD when coexpressed in *N. benthamiana*, indicating that these effectors may interfere with cell death signaling. The *P. syringae* effector AvrPto is recognized by tomato kinase Pto and its cognate R protein Prf (Tobias et al., 1999). Transient expression of AvrPto and Pto trigger HCD in *N. benthamiana*, which carries a Prf homolog (Scofield et al., 1996). Another *P. syringae* effector AvrPtoB interferes with Pto-mediated recognition of AvrPto due to its E3-ligase activity (Abramovitch, 2003; Abramovitch et al., 2006). As expected, agroinfiltration of AvrPto and Pto induced strong HCD whereas coexpression of AvrPtoB did not (**Figure 4B**). We identified only one *Psa* effector, HopQ1, that suppressed AvrPto/Pto-induced HCD. In addition, since some *Psa* effectors induced HCD in *N. benthamiana* (**Figure 2**), we conducted an agroinfiltration assay to test if other *Psa* effectors have the ability to suppress *Psa* effector-induced HCD. Among 8 tested, 5 effectors showed suppression activity on *Psa* effector-induced HCD (**Figure 4C**). Interestingly, HopQ1 suppressed HCD triggered by multiple *Psa* effectors whereas AvrB4-1, HopF4b, HopAR1, and HopA1 suppressed HCD induced by one or two effectors. None of the *Psa* effectors were able to suppress HopAM1-1 or HopZ5-triggered HCD (**Figure 4C**). Overall, these results illustrate the specificity of HCD suppression by multiple *Psa* effectors.

### HopD1 Suppresses PAMP-Induced Inhibition of *Pto* DC3000-Induced Hypersensitive Response in *N. benthamiana*

**FIGURE 5:**
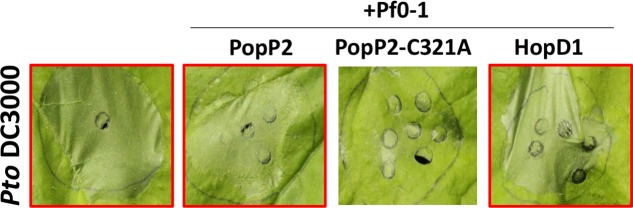
The *Psa* effector HopD1 interferes with *Pf* Pf0-1(T3S)-induced suppression of *Pto* DC3000-triggered HCD. Leaves from 4 to 5 week-old *N. benthamiana* plants were infiltrated with *Pf* Pf0-1(T3S) carrying *popP2*, *popP2-C321A* or *hopD1* (2 × 10^7^ CFU/mL) 8 h prior to *Pto* DC3000 (3 × 10^8^ CFU/mL) infection. *Pto* DC3000-triggered cell death was scored and photographed at 48 h post *Pto* DC3000 infection.

Many bacterial effectors were shown to interfere with PTI to enhance bacterial virulence. PTI triggered by a non-pathogenic bacterial strain can suppress ETI-associated HR elicited by a subsequent infiltration of another bacterial strain (Oh and Collmer, 2005; Crabill et al., 2010). The presence of a PTI-suppressing effector in the non-pathogenic first strain can suppress PTI sufficiently to allow the second ETI-triggering strain to trigger an HR (Le Roux et al., 2015). *Pto* DC3000 delivers effectors via the T3S and induces HCD in *N. benthamiana*. Activation of PTI prior to *Pto* DC3000 infection inhibits effector secretion by the T3S, resulting in significantly reduced HR triggered by *Pto* DC3000. We performed a PTI inhibition assay by delivery of each *Psa* effector from *P. fluorescens* Pf0-1(T3S) prior to *Pto* DC3000 infection in *N. benthamiana* (Le Roux et al., 2015). As shown previously, delivery of the wild-type *Ralstonia solanacearum* effector PopP2 from Pf0-1(T3S) interfered with Pf0-1(T3S)-mediated inhibition of *Pto* DC3000-induced HCD (**Figure 5** and Supplementary Figure S5) (Le Roux et al., 2015). In contrast, the enzymatically inactive PopP2-C321A variant did not affect Pf0-1(T3S)-mediated inhibition of Pto DC3000-induced HCD. Among 35 *Psa* effectors tested, we found that only HopD1 showed robust PTI suppression activity in our assay (**Figure 5** and Supplementary Figure S5). However, this result does not exclude the possibility that other *Psa* effectors carry PTI-suppression activity.

## Discussion

In our study, we aimed to investigate functions of T3S effectors from two economically important strains of *P. syringae* pv. *actinidiae*, a causal agent of bacterial canker in kiwifruit. To better understand the functions of *Psa* T3Es, we generated a library comprising 48 cloned effectors from *Psa* V13 and LV5 strains, characterized their subcellular localization and showed that *Psa* effectors are localized at a diverse range of cellular compartments. By using agroinfiltration, we identified an unusually large number of effectors that induce HCD in *Nicotiana* spp. The requirement of SGT1 for HCD induced by some *Psa* effectors was demonstrated using a VIGS assay. Moreover, we showed that multiple *Psa* effectors suppress HCD. Finally, it was demonstrated that HopD1 interferes with PTI. Taken together, we conclude that multiple *Psa* effectors modify host immune responses via various mechanisms.

### Diverse Localization of *Psa* T3Es Implicates Distinct Functions within the Host Cell

Localization of a T3E *in planta* provides an indication of the host target protein location. For example, several plasma membrane-localized T3Es including AvrRpm1, AvrB, and AvrRpt2 target membrane-associated RIN4 while nuclear-localized T3E PopP2 binds its corresponding NLR, RRS1, inside the nucleus (Nimchuk et al., 2000; Axtell and Staskawicz, 2003; Deslandes et al., 2003). Through our large-scale screening for the sub-cellular localization of transiently expressed *Psa* T3Es, we concluded that the nucleus, cytoplasm and plasma membrane are major target compartments for *Psa* T3Es. *Psa* T3Es of a multitude of sizes that localize to the nucleus make up a large proportion of the T3Es (23/44). Proteins between 90 and 110 kDa were found to passively diffuse through nuclear pores (Wang and Brattain, 2007). This may be one of the reasons for nucleocytoplasmic localization of a large number of effectors. Notably, since the *in planta* subcellular localization was assessed by transient expression of effector-YFP proteins, we cannot rule out the possibility that effector localization may be affected by over-accumulation of proteins or C-terminally tagged YFP. Nonetheless, it appears that *Psa* effectors localized in diverse cell compartments.

Previously, HopZ3 from *P. syringae* pv. *syringae* B728a (approximately 76 kDa including the YFP tag; 72% amino acid identity to *Psa* HopZ3) showed a nucleocytoplasmic localization to target MPK3 and MPK6, while HopBB1 from *P. syringae* pv. *mori* 301020 (approximately 60 kDa including the YFP tag; 93% amino acid identity to *Psa* HopBB1-2) was localized in the nucleus to target TCP14 and JAZ3 for degradation, despite not possessing a specific nuclear localization signal (Lewis et al., 2014; Yang et al., 2017). However, a nuclear localization may be critical to effector function with studies showing that several nucleus-localized T3Es induce transcriptional reprogramming in the host cell (Nissan et al., 2006; Kay and Bonas, 2009). On the other hand, many PRRs and their associated proteins involved in perception of PAMPs or activation of PTI are localized at the plasma membrane. Thus, many T3Es target these PTI components at the plasma membrane once delivered into the host cell (Kim et al., 2005; Zhou et al., 2014). This is consistent with our finding that some *Psa* T3Es are localized at the cell periphery. It would be interesting to further investigate if the *Psa* T3Es localized at the cell periphery enhance bacterial virulence by targeting novel PTI components.

A future application of findings from our study will be to examine *Psa* T3E function in relation to localization. Promising candidates for this could be HopAZ1 or HopAV1 that both appear to target the host cell cytoskeleton. HopZ1a and HopW1 are two known T3Es from *P. syringae* that target the cytoskeleton to disrupt PTI (Lee et al., 2012; Kang et al., 2014). Notably, a HopAZ1 homolog from *P. savastanoi* was recently reported to suppress both ROS production and callose deposition, critical markers of PTI (Matas et al., 2014). *hopAZ1* is present in all sequenced strains of *Psa* and appears to be a ‘*Psa* core effector’ (McCann et al., 2013). Interestingly, *hopAZ1* was reported to have been independently acquired multiple times into the T3E repertoire of pathogens of hazelnut, *P. syringae* pv. *avellenae* (O’Brien et al., 2012). These strains that cause hazelnut decline disease are from two different phylogroups of *P. syringae* strains, suggesting that *hopAZ1* is closely associated with a gain of virulence in this woody host. Considering the subcellular localization of HopAZ1, identification of its interacting proteins *in planta* that also localize to the cytoskeleton could help reveal the role that HopAZ1 plays in promoting *Psa* virulence.

We note several differences for subcellular localization between *Psa* effectors and other known *Pseudomonas* effector homologs. HopM1 from *Pto* DC3000 is known to target AtMIN7 in Arabidopsis and destabilize AtMIN7 within the *trans*-golgi network and early endosome (Nomura et al., 2011). However, *Psa* HopM1 localized to the chloroplast in *N. benthamiana.* As the *Pto* DC3000 and *Psa* homologs of HopM1 share only 66.9% amino acid sequence identity, it is plausible that they target different host proteins *in planta*. However, previous findings have highlighted an alternative possibility since HopM1 was found to suppress SA-mediated immunity (DebRoy et al., 2004) as well as interact with 14-3-3 proteins to mediate pathogen virulence *in planta* (Lozano-Duran et al., 2014). This is particularly interesting since SA synthesis is primarily localized within the chloroplast and exported out by the multidrug and toxin extrusion-like transporter, EDS5, and 14-3-3 proteins have been associated with proteins targeted to the chloroplast (Sehnke et al., 2000; Serrano et al., 2013). Taken together, different localization of T3E homologs suggests that diversity of effector functions and targets may exist even in situations where effectors share significant homology.

### Evidence of HCD-Inducing and -Suppressing *Psa* Effectors Support Pathogen Effector Interplay and Evolutionary Diversification

Multiple *Psa* effectors and their homologs from other *P. syringae* strains were compared for their HCD-inducing activity and, despite high amino acid sequence identity, a few effector homologs (HopAA1 and HopR1) showed different activities in *Nicotiana* spp. (**Table 2**). On the other hand, a HopF homolog from *Psa* V13 and *Pto* DC3000 showed similar HCD-inducing activity in *N. tabacum* despite relatively low amino acid identity. An R protein-mediated recognition for an effector can occur by direct interaction or indirectly through ‘guardee’ or ‘decoy’ molecules which interact with the effector (Jones and Dangl, 2006; van der Hoorn and Kamoun, 2008). Loss of interaction between the effector and its host target (R protein or guardee/decoy) may cause failure of effector recognition and associated cell death (Jones and Dangl, 2006; van der Hoorn and Kamoun, 2008). The HCD-inducing activities for HopAA1 (*Pto* DC3000) and HopR1 (*Psa* V13) may be lost in their corresponding homologs due to evolutionary pressure on the effector active site or recognition determinant to evade recognition while retaining virulence contribution. HopF4b (*Psa* HopF2), meanwhile, is a member of a wide-spread family of ADP-ribosyltransferases present in *P. syringae* strains with extensive genetic diversity (Lo et al., 2016). *Psa* possesses several effectors which share significant amino acid similarity with HopF2: HopF1, HopF4b, HopX3 (a novel member of the HopF family), HopBB1-1, and HopBB1-2. Among these, HopX3, HopBB1-1, and HopBB1-2 are located on the same pathogenicity island, termed the exchangeable effector locus (EEL), sites of significant genome variation between different *Psa* strains (McCann et al., 2013). This suggests that this family of effectors is under evolutionary pressure and may be a case study in evolutionary functional diversification. Unfortunately, mechanisms of cell death triggered by these effectors are still poorly understood. It would be interesting for future work to identify the recognition mechanisms and to search for the sequence variation in the interacting motifs responsible for variation in the HCD phenotype.

Several *Psa* effectors were shown to suppress effector-triggered HCD in this study (**Figure 4**). There are a few hypotheses on how effectors suppress ETI: (1) they share a common target with HCD-eliciting effectors and undergo biochemical processes to antagonistically interfere with the recognition, e.g., AvrRpm1/AvrRpt2/HopF2 (Kim et al., 2005; Wilton et al., 2010) and AvrPto/AvrPtoB (Abramovitch, 2003); (2) they may interfere with the downstream signaling of defense responses triggered by the effectors, e.g., HopF2 (Wang et al., 2010) and HopE1 (Guo et al., 2016); (3) they can interfere with the HCD-eliciting effectors through direct interaction, e.g., HopZ3/AvrB (Lee et al., 2015); or (4) they may suppress *Agrobacterium*-mediated transient protein expression in *N. benthamiana*, e.g., HopQ1 (Adlung and Bonas, 2017). Indeed, *Psa* HopQ1 suppressed most effector-triggered HCD tested in this study but notably failed to suppress HCD triggered by HopZ5, HopT1 and HopAM1-1. It would be interesting if HopZ5, HopT1 and/or HopAM1-1 affect HopQ1-mediated cell death suppression activity. Apart from HopQ1, other *Psa* effector-mediated HCD suppression activities were specific to one or two HCD-eliciting effectors (**Figure 4**). Nevertheless, several *Psa* effectors could suppress *Psa* effector-triggered HCD in *N. benthamiana*. Further research on these specific suppression mechanisms would help reveal the mechanism of ETI/HCD-suppression specificity.

### From HCD-Triggering Effectors to Developing Resistance in Kiwifruit

Not all HCD triggered by agroinfiltration of effectors may be due to activation of immune responses (Vinatzer et al., 2006; Wroblewski et al., 2009). For instance, HopT1-1 triggered HCD but does not appear to be associated with significant growth restriction of virulent bacteria in *N. benthamiana* (Wei et al., 2007; Wroblewski et al., 2009). Conversely, *P. syringae* pv. *tomato* DC3000 lacking *hopQ1-1* can cause disease symptoms in the non-host *N. benthamiana* but agroinfiltration of HopQ1-1 does not result in significant cell death (Wei et al., 2007; Wroblewski et al., 2009; Adlung and Bonas, 2017). Utilization of VIGS to suppress T3E-triggered cell death can offer clues about the downstream mechanism of effector-triggered HCD. The requirement of known immunity-regulator genes would suggest that the effector-triggered HCD is an immune response (Vinatzer et al., 2006). Wei et al. (2007), using VIGS, confirmed that *SGT1* was required for cell death triggered by *Pto-*delivered HopQ1-1. Similarly, the *Psy*B728a effector, HopAA1, partially contributes to growth restriction of this strain on *N. benthamiana* and requires *EDS1* for its HCD development (Vinatzer et al., 2006).

In addition to HopZ5 (Jayaraman et al., 2017), *SGT1* is at least partially required for 5 *Psa* T3Es: HopAM1-1, HopAE1, HopT1, HopW1, and HopAB3. *hopAM1-1* is unique to *Psa* V13 while *hopT1*, *hopW1*, and *hopAB3* are unique to *Psa* LV5. *hopAE1* alleles are present in both *Psa* strains. Interestingly, HopAM1 from *Pto* DC3000 is associated with resistance in Arabidopsis accession Bur-0 (Iakovidis et al., 2016), while HopAB3, a homolog of AvrPtoB from *Pto*DC3000, is an avirulence effector in tomato (Lin et al., 2006). Cell death triggered by HopX1, AvrE1, and HopR1 were largely unaffected by *SGT1*-silencing suggesting limited involvement in immune responses. Support for this notion comes from previous observations suggesting that HopX1, AvrE1, and HopR1 trigger necrosis rather than HR (Lorang and Keen, 1995; Badel et al., 2006; Nimchuk et al., 2007; Kvitko et al., 2009). Further characterization of *Psa* T3Es in *N. benthamiana* through gene knockouts in *Psa* would be useful for identification of avirulence effectors with a view to subsequently identify cognate *R* genes. Based on our results, the strongest avirulence gene candidates would be HopW1, HopAB3, HopZ5, HopAM1-1, and HopAE1.

Identification of *R* genes involved in the recognition of effectors is challenging and laborious. Recently, however, two advances in the field have facilitated this process. Firstly, Brendolise et al. (2017) have developed a hairpin-RNAi library targeting NLRs in *N. benthamiana* that allows for rapid identification of NLRs required for the recognition of an effector. They have demonstrated that the hairpin library is not only efficient at identifying the sensor NLR involved (Prf in the case of AvrPto) but also helper NLRs as well. This system is highly amenable for our purposes and multiple NLRs may be identified for cloning and downstream analyses without first mapping the resistance loci involved. The second technology is the development of RenSeq (Resistance gene enrichment sequencing) – a tool that capitalizes on the enrichment of specific NLR sequence(s) in a screened population of disease resistant plants (Jupe et al., 2013). This technology uses NLR enrichment and resequencing/reannotation to identify resistant alleles of NLRs even in crop plants that are still in the early stages of study. RenSeq and its derivative technologies have been used to identify candidate NLRs for multiple crop plants, including potato, tomato, and wheat (Jupe et al., 2013; Steuernagel et al., 2016; Witek et al., 2016). Application of RenSeq to identify candidate NLRs in tobacco and *N. benthamiana* would accelerate the identification of NLRs that recognize *Psa* effectors and, ultimately, the development of *Psa*-resistant kiwifruit.

## Materials and Methods

### Bacterial Materials

*Escherichia coli* DH5α was used to clone and maintain effector constructs. *Agrobacterium tumefaciens* AGL1 was used for transient transformation of *N. benthamiana* and *N. tabacum* leaf cells. *E. coli* DH5α and *A. tumefaciens* AGL1 were cultured in low salt L-media with appropriate antibiotics at 37 and 28°, respectively. The final concentrations of antibiotics used for bacterial cultures were 100 μg/ml for ampicillin and 50 μg/ml for kanamycin.

### Plant Materials

*Nicotiana tabacum* Wisconsin 38 (W38) and *N. benthamiana* were grown for 4–5 weeks in a controlled plant growth room in long-day conditions (24°C, 16 h light/8 h dark).

### *Psa* Effector Library Cloning Using Golden Gate Assembly

*Psa* strains genomic DNA was extracted using Thermo gDNA extraction kit (GeneJET^TM^, Thermo). All effector sequences were extracted from the published *Psa* genome database (McCann et al., 2013). Briefly, each effector sequence was divided into 1–1.5 kb modules based on length and presence of internal *Bsa*I or *Sma*I restriction enzyme recognition sequences. *Psa* effector modules were PCR-amplified with flanking *Bsa*I site-containing primers using high-fidelity polymerase (Phusion HiFi, Thermo). Amplified PCR products were ligated with *Sma*I-digested entry vector pICH41021. Ligated constructs were verified by *Bsa*I digestion and Sanger sequencing. If present, internal *Bsa*I sites in effector modules were removed by site-directed mutagenesis, as per instructions for the QuickChange II site-directed mutagenesis kit (Agilent, New Zealand). Chaperones for effectors, where present, were amplified with and cloned into the first module and were used exclusively for *Pseudomonas*-delivery constructs. Modules starting with the first codon of the effector coding sequence alone were used for *Agrobacterium* constructs. For *Pseudomonas*-delivery, effectors we cloned into the broad host-range vector pBBR1MCS5B:*avrRps4_pro_* with a C-terminal 6xHA tag using the Golden Gate cloning method (Engler et al., 2008) as described previously (Jayaraman et al., 2017). For agroinfiltration and construction of the *Agrobacterium*-expression library, effectors were cloned into the Golden Gate compatible binary vector pICH86988 under the CaMV 35S promoter and TMV Ω leader and with a C-terminal YFP tag using the Golden Gate assembly method (Engler et al., 2008).

### *Psa-*Triggered HR and *Pseudomonas*-Delivery of *Psa* Effectors

Broad host-range plasmid constructs of each *Psa* effector were mobilized from *E. coli* DH5α to *P. fluorescens* Pf0-1(T3S) strains (Thomas et al., 2009) by triparental mating using *E. coli* HB101 (pRK2013) as a helper strain. *Psa* V13, *Psa* LV5, or Pf0-1(T3S) carrying empty vector or *Psa* effector constructs, were streaked from glycerol stocks onto King’s B plates with antibiotic selection and grown for 2 days at 28°C. Bacteria were then harvested from plates, resuspended in 10 mM MgCl_2_ and diluted to required OD_600_. Infiltrations were carried out on fully expanded leaves of 4- to 5-week-old *N. benthamiana* with a blunt end syringe. For assessing HR, *Psa* V13, *Psa* LV5 or *Pto* DC3000 (OD_600_ = 0.2; 1 × 10^8^ CFU/mL), or Pf0-1(T3S) (OD_600_ = 0.6; 3 × 10^8^ CFU/mL) were infiltrated in patches on three *N. benthamiana* leaves (replicates). HR was assayed visually at 1 day post infiltration (dpi) (*Psa*/*Pto*) or 2–3 dpi (Pf0-1). For assessing PTI suppression, the method was adapted from that described previously (Le Roux et al., 2015). Briefly, 12 h after Pf0-1 infiltration (for non-HR-triggering *Psa* effectors), *Pto* DC3000 (OD_600_ = 0.03; 2 × 10^7^ CFU/mL) was infiltrated in an overlapping area of the leaves. *Pto* DC3000-triggered tissue collapse was scored at 3 dpi. PTI suppression experiments were conducted four times with effectors demonstrating full suppression of *Pto* DC3000-triggered tissue collapse in all three reps in at least three out of four experiments scored as strong suppressors of PTI.

### *Agrobacterium*-Mediated Transient Expression

*Agrobacterium tumefaciens* AGL1 strain was transformed with binary vector effector constructs by electroporation (2.2 kV/6ms/Bio-Rad). Transformed cells were grown on L-agar plates with selective antibiotics for 2 days then inoculated and grown in liquid L-media with selective antibiotics. Overnight grown cultures were centrifuged at 5000 rpm for 4 min, resuspended in agroinfiltration solution (10 mM MgCl_2_/10 mM MES) and adjusted at OD_600_ = 0.4 before leaf infiltration with a blunt end syringe.

### Immunoblot Analysis

*Nicotiana benthamiana* leaves were infiltrated with a mixture of *Agrobacterium* AGL1 harboring the binary vector effector construct (OD_600_ = 0.4) and the silencing suppressor P19 (OD_600_ = 0.1). Five leaf disks (8 mm in diameter) were harvested from the infiltrated patch at 2 days post infiltration and snap frozen in liquid nitrogen. Samples were ground in Laemmli protein loading buffer (Tris-Cl pH 6.8 250 mM, SDS 8%, Bromophenol blue 0.1%, Glycerol 40% and DTT 100 mM) and boiled for 10 min. Total protein extracts were separated by SDS-PAGE, transferred into PVDF membrane (Sigma–Aldrich) and probed with anti-GFP-HRP conjugated antibodies (Santa Cruz Biotech). Super signal West Pico Chemiluminescent sensitivity substrate (Thermo Fisher) and Super signal West Pico Maximum sensitivity substrate (Thermo Fisher) were used for detection.

### Virus-Induced Gene Silencing (VIGS)

Short specific fragments (∼400 bp) from *NbSGT1, NbNDR1*, and *NbEDS1* were designed using the Solgenomics VIGS tool (vigs.solgenomics.net). These fragments were PCR-amplified with primers introducing 5′ end *Eco*RI and 3′ end *Xho*I sites and were cloned into the pTRV2 vector between *Eco*RI and *Xho*I sites (Liu et al., 2002). Two week-old *N. benthamiana* seedlings were infiltrated with a mixture of *A. tumefaciens* AGL1 harboring pTRV1 (OD_600_ = 0.5) and pTRV2 : gene to be silenced (OD_600_ = 0.5) into cotyledons and grown for a further 4–5 weeks (Liu et al., 2002) in short day conditions (22°C, 11 h light/13 h dark). Gene silencing was confirmed by semi-quantitative amplification of the silencing target gene. Total RNA of *N. benthamiana* was extracted from one leaf disk (8 mm in diameter) using the Tri-reagent RNA extraction method (Ambion). cDNA was synthesized with Maxima kit (Thermo) following the manufacturer’s instruction and was used as template to amplify silenced genes with specific primers and Prime Taq polymerase (GenetBio).

### Confocal Microscopy Analysis

*Agrobacterium tumefaciens* AGL1 harboring each *Psa* effector C-terminally tagged with YFP was infiltrated in 4-week-old *N. benthamiana* leaves and YFP signal was detected with a Carl Zeiss LSM700 confocal microscope at 2 days post infiltration. YFP fluorescence was excited at 488 nm with a 20 mW Argon laser and captured by the confocal channel in the emission range 500–530 nm. Images were processed using ImageJ software.

## Author Contributions

SC, JJ, CS, and KS conceived and designed the study. SC, JJ, CS, and KS carried out the experiments. H-JP, HP, and S-WH provided materials and related information. SC, JJ, and KS analyzed and interpreted the data. SC, JJ, and KS prepared the manuscript.

## Conflict of Interest Statement

The authors declare that the research was conducted in the absence of any commercial or financial relationships that could be construed as a potential conflict of interest.
