# Supplementary material for: Pseudomonas syringae pv. actinidiae Type III Effectors Localized at Multiple Cellular Compartments Activate or Suppress Innate Immune Responses in Nicotiana benthamiana
Source: Front Plant Sci. 2017 Dec 20;8:2157. doi: 10.3389/fpls.2017.02157 (PMC5742410; doi:10.3389/fpls.2017.02157)
Supplement: Supplementary file 1 [file Presentation_1.PDF]

Supplementary Material

Pseudomonas syringae pv. *actinidiae* type III effectors localized at multiple cellular compartments activate or suppress innate immune responses in *Nicotiana benthamiana*

Sera Choi [†], Jay Jayaraman[†], Cécile Segonzac, Hye-Jee Park, Hanbi Park, Sang-Wook Han and Kee Hoon Sohn*

[†]These authors have equally contributed to this work

*** Correspondence:** Kee Hoon Sohn: khsohn@postech.ac.kr

The Supplementary Material for this article includes 1 table and 5 figures

Table S1. Modules of Type-III secreted effectors and their overhangs for golden gate cloning generated in this study

Psa effectors	No. of modules	Module 1 (pICH41021)	Module 2 (pICH41021)	Module 3 (pICH41021)	Module 4 (pICH41021)	Module 5 (pICH41021)	Module 6 (pICH41021)
AvrPto5	1	AATG/CGAA	N/A	N/A	N/A	N/A	N/A
HopS2	1	AATG/CGAA	N/A	N/A	N/A	N/A	N/A
HopF4b (HopF2)	1	AATG/CGAA	N/A	N/A	N/A	N/A	N/A
HopAF1	1	AATG/CGAA	N/A	N/A	N/A	N/A	N/A
HopAZ1	1	AATG/CGAA	N/A	N/A	N/A	N/A	N/A
HopH1	1	AATG/CGAA	N/A	N/A	N/A	N/A	N/A
HopAW1	1	AATG/CGAA	N/A	N/A	N/A	N/A	N/A
AvrRpm1	1	AATG/CGAA	N/A	N/A	N/A	N/A	N/A
HopAY1 (V13)	1	AATG/CGAA	N/A	N/A	N/A	N/A	N/A
HopAH1	1	AATG/CGAA	N/A	N/A	N/A	N/A	N/A
HopAI1 (V13)	1	AATG/CGAA	N/A	N/A	N/A	N/A	N/A
HopAM1-1	1	AATG/CGAA	N/A	N/A	N/A	N/A	N/A
HopBB1-2	1	AATG/CGAA	N/A	N/A	N/A	N/A	N/A
HopY1	1	AATG/CGAA	N/A	N/A	N/A	N/A	N/A
AvrD1	1	AATG/CGAA	N/A	N/A	N/A	N/A	N/A
AvrB4-1	1	AATG/CGAA	N/A	N/A	N/A	N/A	N/A
HopAO2	1	AATG/CGAA	N/A	N/A	N/A	N/A	N/A
HopZ5	1	AATG/CGAA	N/A	N/A	N/A	N/A	N/A
HopN1	1	AATG/CGAA	N/A	N/A	N/A	N/A	N/A
HopX3	1	AATG/CGAA	N/A	N/A	N/A	N/A	N/A
HopZ3	3	AATG/TTTC	GAAA/GGAT	ATCC/CGAA	N/A	N/A	N/A
HopQ1	2	AATG/GTTG	CAAC/CGAA	N/A	N/A	N/A	N/A
HopI1	1	AATG/CGAA	N/A	N/A	N/A	N/A	N/A
HopD1	3	AATG/GCGT	ACGC/GTTG	CAAC/CGAA	N/A	N/A	N/A
HopM1	3	AATG/CAGT	ACTG/GTAC	GTAC/CGAA	N/A	N/A	N/A
HopAU1	3	AATG/CTGC	GCAG/ACTG	CAGT/CGAA	N/A	N/A	N/A
HopAE1	4	AATG/CTTC	GAAG/AGTG	CACT/GCGC	GCGC/CGAA	N/A	N/A
HopAV1	4	AATG/ATGC	GCAT/TGGC	GCCA/CAGG	CCTG/CGAA	N/A	N/A
HopAS1	5	AATG/GCGT	ACGC/CCTG	CAGG/ACAG	CTGT/CGAA	CTGT/CGAA	N/A
AvrE1	6	AATG/GCGT	ACGC/GAAT	ATTC/CGCC	GGCG/CTTC	GAAG/ATTG	CAAT/CGAA
HopR1	6	AATG/TCTG	CAGA/ATGT	ACAT/GTCG	CGAC/CGGC	GCCG/TGTT	AACA/CGAA
HopBN1	1	AATG/CGAA	N/A	N/A	N/A	N/A	N/A
HopAB3	2	AATG/TGCC	GGCA/CGAA	N/A	N/A	N/A	N/A
HopAF1-2	1	AATG/CGAA	N/A	N/A	N/A	N/A	N/A
HopAG1	2	AATG/TGTC	GACA/CGAA	N/A	N/A	N/A	N/A
HopAY1 (LV5)	1	AATG/CGAA	N/A	N/A	N/A	N/A	N/A
HopE1	1	AATG/CGAA	N/A	N/A	N/A	N/A	N/A
HopO1	1	AATG/CGAA	N/A	N/A	N/A	N/A	N/A
HopS1	1	AATG/CGAA	N/A	N/A	N/A	N/A	N/A
HopT1	1	AATG/CGAA	N/A	N/A	N/A	N/A	N/A
HopW1	2	AATG/GGCA	TGCC/CGAA	N/A	N/A	N/A	N/A
HopA1	1	AATG/CGAA	N/A	N/A	N/A	N/A	N/A
HopF1	1	AATG/CGAA	N/A	N/A	N/A	N/A	N/A
HopAR1	1	AATG/CGAA	N/A	N/A	N/A	N/A	N/A
HopX1	1	AATG/CGAA	N/A	N/A	N/A	N/A	N/A
HopX2	1	AATG/CGAA	N/A	N/A	N/A	N/A	N/A
HopAA1	1	AATG/CGAA	N/A	N/A	N/A	N/A	N/A
HopAI1 (LV5)	2	AATG/CGAA	N/A	N/A	N/A	N/A	N/A

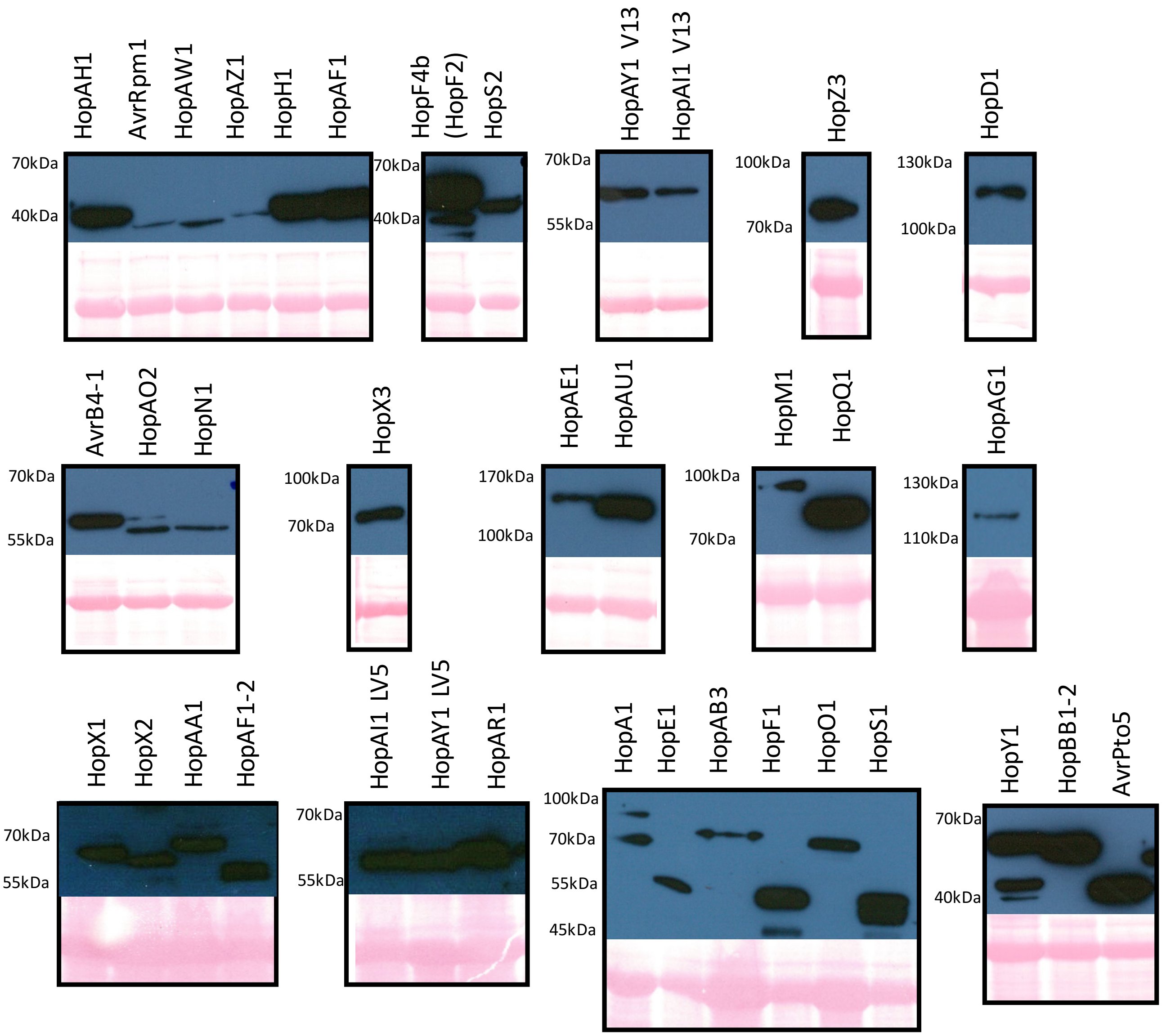

Figure S1. *Psa* effectors are expressed *in planta*. Detection of YFP-tagged *Psa* effectors from transient expression in *N. benthamiana* plants. *Agrobacterium tumefaciens* AGL1 harboring *Psa* effectors were co-infiltrated at OD_{600}=0.4 with plant viral suppressor of gene silencing P19 at OD_{600}=0.1 into leaves of 4-5 week-old *N. benthamiana*. Six 8mm diameter leaf discs from the infiltrated patches were harvested at 2 days post infection (dpi). Protein was extracted by applying an equal volume of 2X Leamli protein loading buffer. Samples were separated by SDS-PAGE, blotted on to PVDF membranes and detected by anti-GFP HRP-conjugated antibody. Proteins of roughly similar sizes were run together and only the visualized protein bands of correct size are shown. Ponceau Red staining of total protein extract for the Rubisco band is shown below each panel and is used as sample loading control.

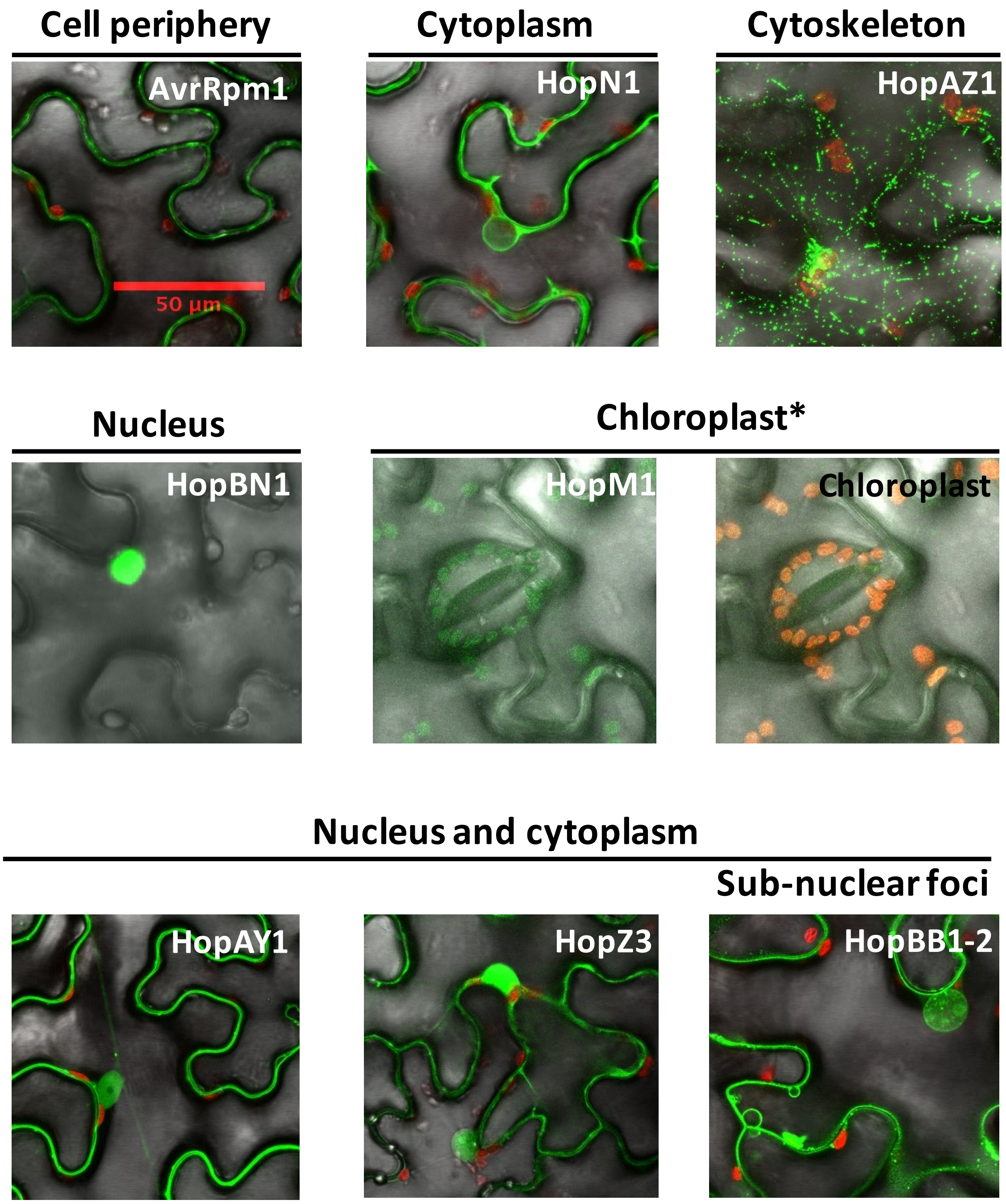

Figure S2. Representative images of *Psa* effector localization in *N. benthamiana* epidermal cells. 4-5 week-old *N. benthamiana* leaves were infiltrated with *Agrobacterium* AGL1 carrying C-terminally YFP-tagged *Psa* effectors for transient protein expression. At 2 dpi, 8mm diameter leaf discs were harvested and viewed using confocal laser-scanning microscopy. YFP fluorescence was excited at 488nm with a 20mW Argon laser and captured in the emission range between 500-530nm. The HopM1 YFP signal was determined by a sum-of-squares Z-projection. Chloroplast auto-fluorescence was detected between 600-680nm.

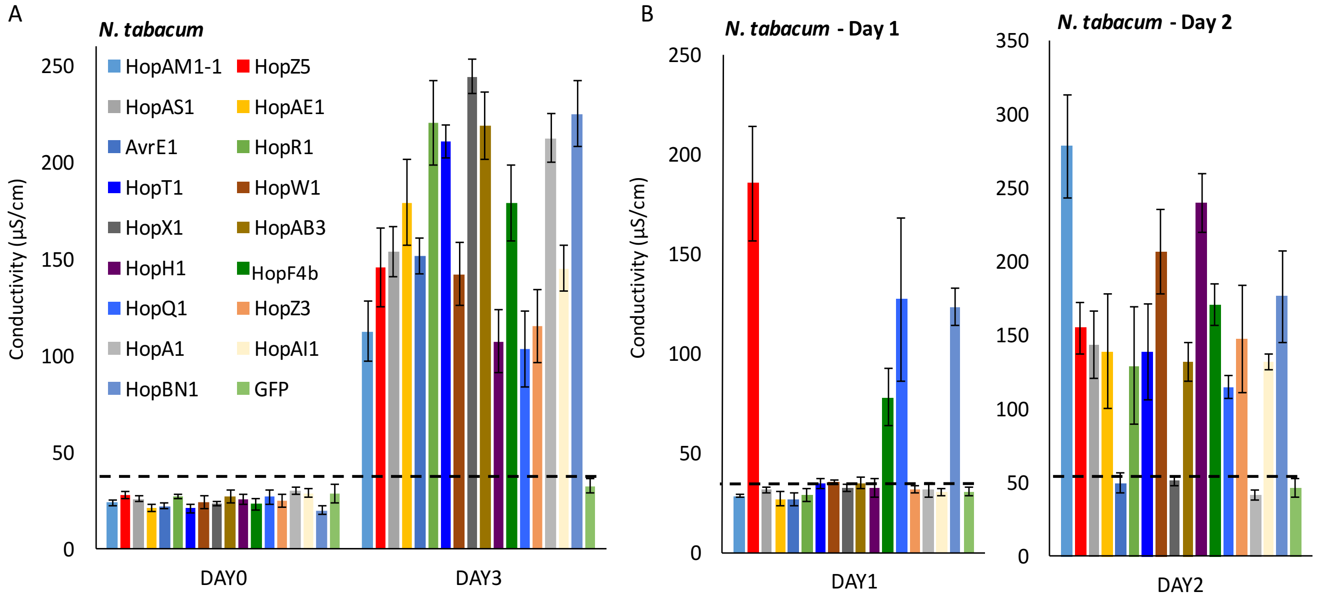

Figure S3. Electrolyte leakage assay of *Psa* effector-triggered HCD in *N. tabacum.* (A) 4-5 week-old *N. tabacum* leaves were infiltrated with *Agrobacterium* AGL1 carrying C-terminally YFP-tagged *Psa* effectors for transient protein expression. For each sample at the indicated time, two 8mm diameter leaf discs were harvested into 2mL of distilled water, shaken for 2 hours and measured for ion conductivity. 6 replicates of each sample were measured. *Psa* effectors that trigger early (strong, ++) HCD are indicated in (B). Error bars indicate standard error of the mean. The horizontal superimposed dashed line indicates the upper limit of ion conductivity for the negative control (GFP).

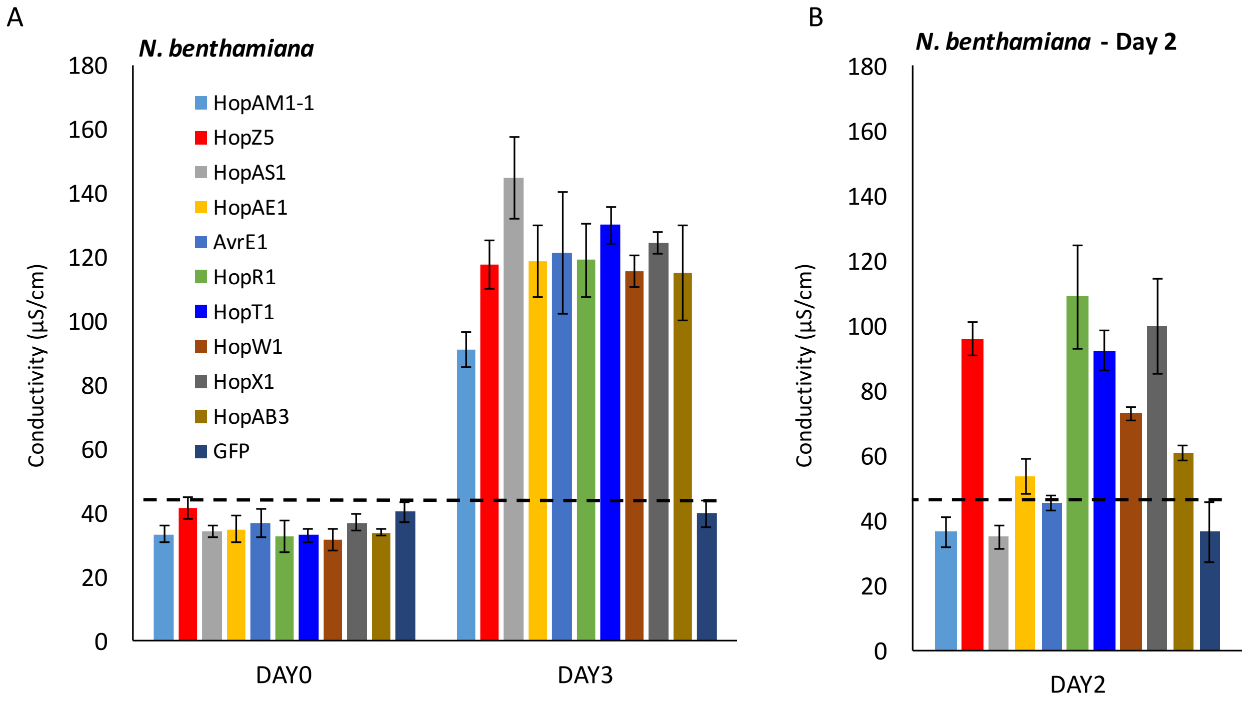

Figure S4. Electrolyte leakage assay of *Psa* effector-triggered HCD in *N. benthamiana*. (A) 4-5 week-old *N. benthamiana* leaves were infiltrated with *Agrobacterium* AGL1 carrying C-terminally YFP-tagged *Psa* effectors for transient protein expression. For each sample at the indicated time, two 8mm diameter leaf discs were harvested into 2mL of distilled water, shaken for 2 hours and measured for ion conductivity. 6 replicates of each sample were measured. *Psa* effectors that trigger early (strong, ++) HCD are indicated in (B). Error bars indicate standard error of the mean. The horizontal superimposed dashed line indicates the upper limit of ion conductivity for the negative control (GFP).

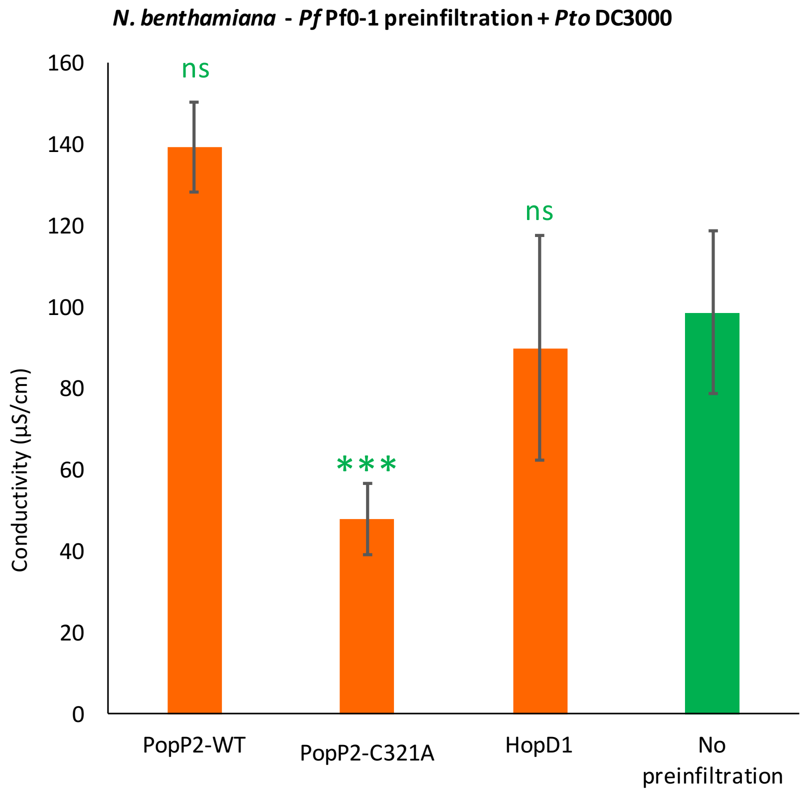

Figure S5. Electrolyte leakage assay for suppression of PTI by *Psa* effector HopD1 in *N. benthamiana.* 4-5-week-old *N. benthamiana* leaves were infiltrated with *Pf* Pf0-1(T3S) carrying C-terminally 6xHA-tagged *popP2*, *popP2-C321A* or *hopD1* ($2x10^7$ CFU/mL) 8 hours prior to *Pto* DC3000 ($3x10^8$ CFU/mL) infiltration. At 48hpi, two 8mm diameter leaf discs were harvested into 2mL of distilled water, shaken for 2 hours and measured for ion conductivity. 6 replicates per sample were measured. Error bars indicate standard deviation of the mean. ***: $p < 0.001$, ns: not significantly different from non-preinfiltrated sample; determined by Student's t-test comparison for each sample (in orange) to non-preinfiltrated sample (in green).
